# Anatomical landmark-guided compartment block in pediatric lateral thoracotomy: implications for the vertebral arch surface

**DOI:** 10.1186/s40981-024-00751-y

**Published:** 2024-10-28

**Authors:** Tomohiro Yamamoto, Marian Mikus

**Affiliations:** 1 Division of Anesthesiology, Niigata University Graduate School of Medical and Dental Sciences, Niigata, Japan; 2 Department of Anesthesiology and Intensive Care Medicine, University Hospital Bonn, Bonn, Germany

To the Editor,

Lateral thoracotomy is commonly used for cardiothoracic surgeries in pediatric patients. In these procedures, peripheral nerve blocks targeting the intercostal nerves at multiple segments can be a key to perioperative anesthesia management. Erector spinae plane block (ESPB) [1, 2], retrolaminar block (RLB) [3], paravertebral block (PVB) [4], and midpoint transverse process to pleura block [5] are examples that target the intercostal nerves proximal to the spinal cord. These compartment blocks are often referred to as 'paravertebral blocks by proxy’ because the primary difference lies in the needle tip position. The local anesthetic spreads into the paravertebral space without the need to advance as deeply as in PVB, yet it produces the same effect [6]. Among these blocks, ESPB and RLB, where local anesthetic is administered to the vertebral arch surface, can be performed quickly and safely using a landmark-guided technique in both pediatric patients, including infants, and adults. Figure 1 and Supplementary Video S1 demonstrate the landmark-guided ESPB/RLB technique performed on a 1-month-old infant (58.7 cm, 4.7 kg) in the lateral position for right diaphragm plication via lateral thoracotomy. Figure 2 and Supplementary Video S2 illustrate the landmark-guided ESPB/RLB procedure on a 6-month-old infant (62 cm, 5.1 kg) in the prone position for thoracoscope-assisted surgery for congenital type A esophageal atresia. As shown in the computed tomography images (Figs. 1 and 2), the distance from the spinous process to the lateral edge of the transverse process at the 4th thoracic spine level in infants is less than 1.5 cm. Therefore, the puncture site for these compartment blocks should be just lateral to the spinous process, approximately half a finger’s width away. In these cases, 7–8 mL of 0.15% ropivacaine was administered. It is crucial to determine the appropriate concentration and dosage of the local anesthetic based on institutional guidelines to avoid exceeding the maximum recommended dose, in particular, infants are considered at higher risk of local anesthetic systemic toxicity due to lower alpha-1-acid glycoprotein concentrations [7]. However, a minimum of 1 mL/kg was used to ensure adequate spread of the anesthetic across multiple segmental levels, based on the author’s experience. In pediatric patients, the local anesthetic spreads along the erector spinae muscle to multiple segmental levels with ESPB/RLB, as demonstrated in the figures and videos.

**Fig. 1:**
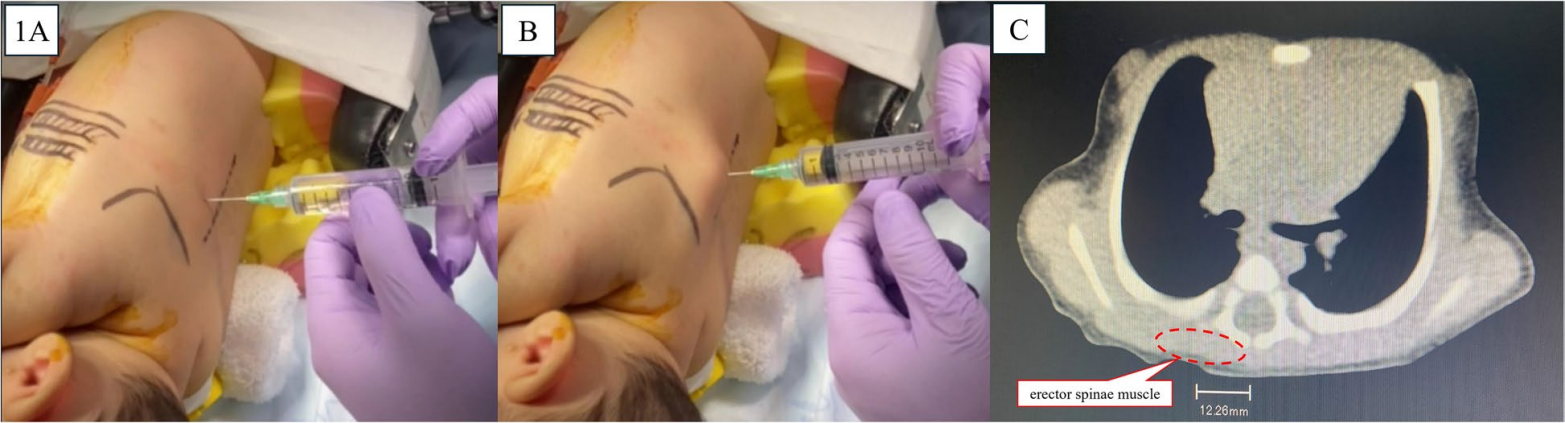
Landmark-guided erector spinae plane block/retrolaminar block performed on a 1-month-old infant. Landmark-guided erector spinae plane block/retrolaminar block was performed in the surgical lateral position in a 1-month-old infant (58.7 cm, 4.7 kg) for right diaphragm plication via lateral thoracotomy. A NIPRO 23G needle (32 mm long) was used. **A** Needle tip at the vertebral arch with 8 mL of 0.15% ropivacaine was administered. **B** After administration, the local anesthetic has spread along the erector spinae muscle to a multi-segmental level, visible by the muscle’s displacement. The black dotted line on the patient’s back indicates the spinous process. **C** The distance from the spinous process to the lateral edge of the transverse process at the 4th thoracic spine level in this infant was less than 1.5 cm in this infant

**Fig. 2:**
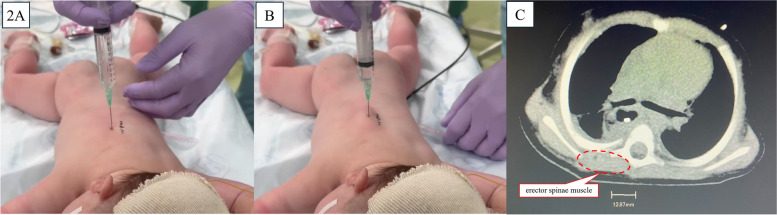
Landmark-guided erector spinae plane block/retrolaminar block performed on a 6-month-old infant. Landmark-guided erector spinae plane block/retrolaminar block was performed in the surgical prone position in a 6-month-old infant (62 cm, 5.1 kg) for thoracoscope-assisted surgery for congenital type A esophageal atresia. A NIPRO 23G needle (32 mm long) was used. **A** Needle tip at the vertebral arch with 7 mL of 0.15% ropivacaine was administered. **B** After administration, the local anesthetic has spread along the erector spinae muscle to a multi-segmental level, visible by the muscle’s displacement. The black dotted line on the patient’s back indicates the spinous process. **C** The distance from the spinous process to the lateral edge of the transverse process at the 4th thoracic spine level in this infant was less than 1.5 cm in this infant

The advancement of ultrasound-guided technology has allowed peripheral nerve blocks to be performed at greater depths [3–5]. However, more complex and time-consuming peripheral nerve blocks may limit their acceptance by surgeons, especially for blocks that can be efficiently performed using landmark-guided methods. This reluctance could prevent patients from benefiting from the analgesic advantages of peripheral nerve blocks. Despite varying opinions on the spread of local anesthetics administered [8–10], ESPB has been reported to be effective in providing analgesia for both adult and pediatric patients undergoing anterior thoracic surgery [1, 2, 11, 12], as well as dorsal to lateral thoracic procedures [13]. Given these considerations, landmark-guided ESPB/RLB offers a valuable alternative, with high feasibility even in challenging surgical positions like lateral and prone.

## Supplementary Information

Additional file 1: Supplementary Video S1: Landmark-guided erector spinae plane block/retrolaminar block performed in the surgical lateral position on a 1-month-old infant (58.7 cm, 4.7 kg) for a right diaphragm plication via a lateral thoracotomy. Once the needle tip reached the vertebral arch, 8 mL of 0.15% ropivacaine was administered. After the local anesthetic was administered, it was visible that it was spread along the erector spinae muscle to a multi-segmental level by pushing up the erector spinae muscle. The black dotted line on the patient’s back is drawn over the spinous process. A NIPRO 23G needle (32 mm long) was used.

Additional file 2: Supplementary Video S2: Landmark-guided erector spinae plane block/retrolaminar block performed in the surgical prone position on a 6-month-old infant (62 cm, 5.1 kg) for a thoracoscope-assisted surgery for congenital type A esophageal atresia. Once the needle tip reached the vertebral arch, 7 mL of 0.15% ropivacaine was administered. After the local anesthetic was administered, it was visible that it was spread along the erector spinae muscle to a multi-segmental level by pushing up the erector spinae muscle. The black dotted line on the patient's back is drawn over the spinous process. A NIPRO 23G needle (32 mm long) was used.

## Abbreviations

ESPB: Erector spinae plane block
RLB: Retrolaminar block
PVB: Paravertebral block
